# Supplementary material for: Are carbon emissions trading and green financial instruments synergistic? -Comprehensive quantitative research based on content analysis
Source: PLoS One. 2024 Mar 7;19(3):e0298601. doi: 10.1371/journal.pone.0298601 (PMC10919601; doi:10.1371/journal.pone.0298601)
Supplement: S1 Data — (ZIP) [file pone.0298601.s001.zip › Data/地级市Z_F工作报告.zip!/║■─╧/║Γ╤⌠╩╨/└┤╫╘╣1⁄2╓┌║┼í╛┬φ┐╦╩2╛▌═°í┐_2019.docx]

各位代表：

现在，我代表市人民政府，向大会报告工作，请予审议，并请各位政协委员和其他列席人员提出意见。

一、2018年工作回顾

2018年，面对错综复杂的国际环境和艰巨繁重的改革发展稳定任务，市政府坚持以习近平新时代中国特色社会主义思想为指导，坚决落实习近平总书记关于湖南工作的重要指示精神，按照李克强总理勉励衡阳争当承接东部产业转移“领头雁”的嘱托，在省委、省政府和市委的坚强领导下，团结和依靠全市人民迎难而上，统筹做好稳增长、促改革、调结构、惠民生、防风险各项工作，保持了经济发展稳中有进的良好态势和社会大局稳定，向高质量发展迈出坚实步伐。

经济运行质量稳步提升。地区生产总值增长8.5%(预计数，下同)。实现一般公共预算收入264.69亿元，增长5.03%;税收收入增长14.62%，税占比达78.1%，同比提高6.54个百分点。三次产业结构优化为10.8∶34.1∶55.1，第三产业占比提高4.4个百分点。高新技术产业增加值增长11%。

创新开放活力明显增强。获批建设国家创新型城市，荣获全省唯一的国家“守信激励创新奖”;获得国省科技奖励11项，每万人有效发明专利拥有量达2件。新引进顺丰集团、杉杉集团、华泰汽车等21家“三类500强”企业，比亚迪、华侨城等重大项目实现当年签约落地、当年开工建设，“营造良好环境、承接产业转移”经验做法获国务院通报表扬。

大城崛起步伐不断加快。成功跻身国家大城市行列，入选国家物流枢纽承载城市，获批湘南湘西承接产业转移示范区，衡阳迎来历史性机遇，正在步入国省重大战略布局“主舞台”。南岳机场通航城市达23个，旅客吞吐量突破80万人次，正朝湘中南地区航空枢纽阔步前行。成功承办省十三运会、省十届残运会，取得金牌总数全省第一的历史佳绩，被誉为“全省最好、全国一流”。

人民生活水平持续改善。13项重点民生实事圆满完成;全体居民人均可支配收入达25879元，增长8.5%，城乡居民收入差距进一步缩小。绿色共享发展成果惠及全市人民，森林覆盖率达48.2%，湘江干流11个监测断面水质全部达到Ⅱ类标准，城区空气质量优良率达86.1%，“衡阳蓝”“湘江绿”成为靓丽名片。

一年来，我们重点做了以下工作：

(一)坚持补短板强弱项，全力打好三大攻坚战。重大风险防范化解有力有效。建立健全偿债应急准备金、债务风险预警、应急以及问责机制，“停缓调撤”一批政府性投资项目;加快推进融资平台公司市场化转型，与华侨城集团合作创新央地国企混改模式，一次性化解市级政府隐性债务82亿元，为政府性平台公司转型发展贡献了衡阳方案;全市债务风险基本可控。严厉打击非法集资，逐步规范地方金融组织发展，金融市场抗风险能力持续增强。脱贫攻坚取得重大突破。实现108个贫困村出列，60979名贫困人口脱贫，祁东县成功脱贫摘帽并得到巩固，全市贫困发生率降到0.99%。完成易地扶贫搬迁8731人，完成自然村通水泥路1228公里，解决17.1万贫困人口饮水安全问题，贫困村电网改造升级全面完成，“社会救助五个一批”做法在全省推广。产业扶贫“四大结合”行动深入开展，油茶产业扶贫经验在全国推介，产业帮扶到户覆盖率达96.3%。成功承办全国“一村一品”产业扶贫经验交流会，常宁市塔山瑶族乡获评全国“一村一品”示范村镇。生态环境保护成效显著。坚决扛起“守护好一江碧水”的政治责任，全力配合中央环保督察“回头看”，强力推进1200余件中央、省环保督察交办的环境问题整改和污染防治攻坚战三年行动计划，扎实开展蓝天、碧水、净土保卫战，201项年度任务全面完成。河长制深入推进，角山、松木污水处理厂即将投运，蒸水城区段入河排污口即将全部封堵，中心城区11个黑臭水体完成整治，13个县级以上集中式生活饮用水水源保护区水质达标率继续保持在100%。工业、燃煤、扬尘等大气污染综合防治全面加强。43个土壤重金属污染场地完成修复治理。第二次全国污染源普查顺利实施。“三线一单”编制工作圆满完成。

(二)坚持重实业兴实体，全力推动高质量发展。工业经济迈向“升级版”。振兴实体经济“3311”工程计划和工业原地倍增计划深入实施，规模工业增加值增长7.5%。实体经济发展质量不断提升，军民融合、有色金属、轨道交通、精细化工、新能源汽车和纺织服装等千亿级产业集群来势喜人。建滔环氧氯丙烷、10万吨离子膜烧碱项目竣工，水口山铜铅锌产业基地30万吨锌、力赛大型锂电池储能(一期)、富士康IDX产业园等项目投产，恒天智电雁城牌新能源客车交付使用，比亚迪智能制造产业园、湘南纺织产业基地、新能源汽车产业、黄金集团高性能铝合金新材料等项目进展顺利，70余家企业开展智能化改造，南岳生物制药获评国家绿色工厂，“衡阳制造”正奋力再次崛起。园区支撑作用进一步增强，省级以上工业园区规模工业增加值占全市规模工业增加值比重达70%以上，松木经开区通过国家循环化改造示范试点园区终期验收。特色农业加快发展。“两区划定”全面完成，粮食总产超过330万吨。新造油茶林14.2万亩、低改垦复17.6万亩，成功承办全国油茶产业发展现场会，发布全省首个茶油地方标准，全国唯一的“国家油茶生物产业基地”落户我市，打造中国油茶第一强市迈出坚定步伐。新增“三品一标”产品认证126个，金甲岭萝卜、衡山红脆桃、衡东黄贡椒荣获国家农产品地理标志登记，“三黄两茶”和特色蔬菜产业被列入“一县一特”省级主导特色产业发展指导目录。常宁市获批全国农村一二三产业融合发展先导区。衡南县获批全国农业社会化服务体系建设试点县。现代服务业持续壮大。社会消费品零售总额增长10.6%。市金融信用信息服务中心服务模式在全国创造衡阳经验;53家企业纳入市重点上市后备企业资源库。白沙物流园投入运营，唯品会衡阳电子信息产业园、衡缘物流中心等项目进展顺利，全国首个工惠驿家线下项目落户我市;完成电商交易额165亿元，增长27%。南岳旅游带动效应明显，工业旅游、乡村休闲游成为新热点，全市共接待游客7016.2万人次，实现旅游总收入630.3亿元，分别增长13.2%、11.2%。

(三)坚持推改革求创新，全力培育发展新动能。重点改革不断深入。以壮士断腕的决心关停并转，关闭非煤矿山11座、中小煤矿8处，清理处置国有“僵尸企业”9家。农业供给侧结构性改革步伐不断加快，农村土地确权登记颁证和农村集体产权制度改革清产核资工作基本完成。“放管服”改革持续深化，“多证合一、一照一码”改革有条不紊，“最多跑一次”改革加速推进，营商环境进一步优化。国企国资改革稳步实施。商事、城管执法、生态环保等领域改革有力有效。创新创业氛围浓厚。大众创业、万众创新深入人心，各类市场主体达29.6万户，实现创业带动就业5.5万人。首个国家级孵化器科创智谷即将投运。新增高新技术企业61家。人才新政有效实施，创新发展平台不断完善，新增院士专家工作站6家、省重点实验室2个;南岳电控获评国家企业技术中心。开放水平明显提升。优化营商环境20条、招商引资30条等政策叠加实施、落地见效，“2018衡州经济发展论坛”“长洽周”等活动成果丰硕，全年签约引进项目122个，总引资额1169.9亿元。五定班列、无水港、公路口岸、综保区稳健运行。95家企业实现外贸业绩“破零倍增”，进出口总额居全省第二，富士康进出口总额继续保持全省单体企业第一。与“一带一路”沿线50余个国家和地区建立经贸往来，国际友好城市总数达8个，衡阳的“朋友圈”越来越大。

(四)坚持抓协调促统筹，全力建设最美地级市。中心城区建管全面提质。科学规划、精美建设、精致管理要求全面落实，全国文明城市创建、新一轮城市总体规划修编等工作稳步开展，历史文化名城保护规划上报省政府审批。陆家新区、滨江新区、来雁新城等片区开发加速推进。城市“双修”稳步实施，“三江六岸”生态绿地建设扎实推进，湘江、蒸水游步栈道建设基本完成，雁栖湖片区综合开发进展顺利，“三塔一院”修缮工程有序铺开，东洲岛绽放新颜。开工棚户区改造1.8万户。城区公厕全面升级到三类以上标准。完成拆违37.4万平方米,在全省率先启动电动摩托车登记管理，市区烟花爆竹禁放扎实推进，中元节文明祭祀蔚然成风，“两站一场”整治成效明显，城市管理水平和文明程度持续提升。立体交通网络健全完善。怀邵衡铁路衡阳段建成通车;大源渡二线船闸主体基本完成，祁东归阳千吨级码头建设稳步推进;二环北路基本建成，东洲湘江大桥即将中跨合龙，比亚迪胶轮有轨电车示范线建设顺利启动;内联外拓的交通网络正由蓝图变成通途。乡村振兴战略全面启动。深入推进农村人居环境综合整治，扎实开展“三清三拆五治”行动。实施1118个村庄规划编制，创建美丽乡村示范村33个，珠晖区金甲梨园入选全国百个乡村旅游景点，衡南县宝盖村入选全国“100个特色村庄”。农村空闲宅基地腾退全面启动，耕地占补平衡扎实开展。二号首长

(五)坚持惠民生促和谐，全力提升群众获得感。全面践行以人民为中心的发展思想，持续加大民生投入，民生支出占一般公共预算支出比重达72.3%。保障体系更加健全。新增城镇就业6.75万人，城镇登记失业率控制在3.5%左右。社会保障扩面提标，城市低保标准由每人每月420元提高到480元，县(市)和城区农村低保由每人每月260元分别提高到320元、480元。机关事业单位养老保险制度改革顺利实施，企业退休人员养老金实现“十四连调”。异地就医住院费用直接结算全面启动，24家定点医院纳入国家异地就医结算平台。改造农村危房8596户。在全省首创“慈惠民生保险项目”。社会事业加快发展。超大班额基本化解，“全面改薄”成效明显，学前教育快速发展，铁一中、市九中完成搬迁，市十七中迁建和市示范性综合实践基地、衡阳幼师高专建设等项目推进顺利。公立医院综合改革有序进行，分级诊疗体系建设不断完善。文化事业欣欣向荣，田汉大奖实现零的突破，电影《一代女魂唐群英》获北欧国际电影节、澳门国际电影节多项提名，《援军明日到达――1944衡阳保卫战》成功立项。质量、民族、宗教、移民、气象、农开、供销工作扎实推进，消防、水文、港澳台、海关、文化市场综合执法工作不断加强，妇女、儿童、老龄事业健康发展。公共治理创新推进。全市安全生产、食品药品安全态势总体平稳。稳妥处置京港澳高速衡阳段“6·29”重大道路交通事故、耒阳“9·1”事件和衡东“9·12”案，举一反三加快完善社会治理体系。石鼓区演武坪社区工作法在全国推广。“雪亮工程”加快建设，“一村一辅警”加速配置，“空中快警”正式组建，“扫黑除恶”经验被中央政法委推介，“云端2018”专项行动荣获全国先进，缉毒战果排名全省前列，“平安衡阳”建设再上台阶。扎实开展信访法治化建设，化解各类矛盾纠纷1.8万余起，办理中央、省移交信访件1500余件，全市进京非访登记同比下降45.6%，信访工作长效机制基本形成。

(六)坚持转作风优服务，全力锻造最强执行力。“互联网+政务服务”一体化平台建设全面启动，政务服务事项网上发布率达100%，依申请类事项网上可办率达75.6%，政务服务工作获全省“五年评比”先进，“12345”政府服务热线连续两年进入全国30强，荣获全国热线“先锋”奖。自觉接受人大及其常委会法律监督和工作监督、政协民主监督以及社会各界的监督，充分采纳各民主党派、工商联和无党派人士意见、建议，办理市人大代表议案2件、建议332件，办理市政协提案524件。提请市人大常委会审议《衡阳市森林防火条例》，出台首部政府规章《衡阳市畜禽规模养殖污染防治管理办法》。政府法律顾问、重大事项报告等制度全面落实。市、县、乡、村四级公共法律服务实体平台实现全覆盖。党风廉政建设和反腐败斗争成果不断巩固，机构编制管理严格规范，行政监督、审计监督、财政监管全面加强。

国防动员和后备力量建设质量提高。国家安全、国防动员、人民防空建设成效突出，优抚安置和民兵预备役建设持续加强，我市成为全省唯一的人防融合建设和宣传教育“五进四化”试点城市，军民融合发展呈现新局面。

各位代表!当前，衡阳加快发展谋篇布局的骨架已经拉开，振翅起飞的势能已经积聚，换道超车的动力正在形成。这些成绩的取得，得益于习近平新时代中国特色社会主义思想的伟大指引，得益于省委、省政府和市委的正确领导，得益于全市人民的团结奋斗，得益于各级人大、政协监督和社会各界人士的关心支持。在此，我代表市人民政府，向全市各族人民，向各民主党派、工商联、各人民团体，向驻衡部队、武警官兵、政法干警和民兵预备役人员，向所有关心支持衡阳发展的各界人士和朋友们，表示诚挚的感谢和崇高的敬意!

同时，我们也必须清醒地看到，经济社会发展仍存在不少短板和困难。一是发展质量还不够高，部分指标未能实现既定目标;经济发展结构还不优、效益还不高，县域经济薄弱，振兴实体经济面临诸多困难。二是城市发展与省域副中心定位还有差距，城市建设品质和管理精细化水平还不够高，城市更新推进缓慢，城镇化水平有待进一步提升，区域协调发展问题不少。三是各类风险矛盾交织，化解政府性债务、防范金融风险、安全生产、生态环保、社会治理等仍存在薄弱环节。四是民生领域存在不少短板，教育、医疗、社保、住房等公共服务供给还不能完全满足群众需求。五是作风建设仍不能完全适应新要求，不担当、不作为、不敢为、不善为等问题不同程度存在。对此，我们一定高度重视，认真研究，切实解决。

二、2019年工作安排

2019年是新中国成立70周年，是全面建成小康社会关键之年。综观国内外形势，经济发展环境更加严峻，不确定性更加凸显，危和机同生并存，但机遇远大于挑战。我国经济长期向好的基本面没有变，支撑高质量发展的要素条件没有变。随着湘南湘西承接产业转移示范区获国家批复建设，随着衡阳被赋予湘南地区中心城市、国家大城市战略定位，随着我市成功获批建设国家创新型城市、入选国家物流枢纽承载城市和国家先进制造业试点示范城市，随着全市若干千亿级产业集群落子布局、“三类500强”企业纷至沓来，衡阳迎来了千载难逢的战略机遇期和换道超车的历史窗口期。站在新的发展起点，我们务必抢抓战略机遇，抢占发展先机，坚定不移办好衡阳的事情，奋力走在全省发展前列，彰显省域副中心城市和承接东部产业转移“领头雁”的使命担当!

2019年政府工作的总体要求是：以习近平新时代中国特色社会主义思想为指导，认真贯彻习近平总书记关于湖南工作的重要指示精神，统筹推进“五位一体”总体布局，协调推进“四个全面”战略布局，坚持稳中求进工作总基调，坚持新发展理念，坚持推动高质量发展，以供给侧结构性改革为主线，深入实施创新引领开放崛起战略，争当承接东部产业转移“领头雁”，持续开展“产业项目建设年”和“基础设施补短板年”活动，全力振兴实体经济，突出打好“三大攻坚战”，扎实推进乡村振兴战略，统筹做好稳就业、稳金融、稳外贸、稳外资、稳投资、稳预期各项工作，加快建设名副其实的省域副中心城市和最美地级市，不断增强人民群众获得感、幸福感、安全感，为全面建成小康社会收官打下决定性基础，以优异成绩庆祝中华人民共和国成立70周年。

2019年全市经济社会发展主要预期目标是：地区生产总值增长8.5%左右，一般公共预算收入增长7.5%左右，固定资产投资增长12%以上，社会消费品零售总额增长11%以上，进出口总额增长18%以上，规模工业增加值增长8%左右，全体居民人均可支配收入增长8.5%左右，全面完成省里下达的节能减排、安全生产等约束性指标任务。

围绕以上目标，要重点做好以下工作：

(一)全力以赴推动高质量发展

坚持质量第一、效益优先，着力抓创新、强产业、扩内需，构建现代经济体系。

推动制造业优化升级。持续推进振兴实体经济“3311”工程计划和工业原地倍增计划，支持白沙绿岛争创国家级军民融合产业示范区，加快打造军民融合、有色金属、新能源汽车、轨道交通、精细化工、纺织服装等千亿级产业集群，新增规模工业企业100家以上。加快完善新一代信息技术、新材料、新能源、生物医药及高性能医疗器械、新型轨道交通装备、输变电高端装备和精密模具等先进制造业产业体系。实施新一轮重点技术改造升级，引导有色金属、盐卤化工、钢管深加工等传统产业提质升级，稳步推进企业优胜劣汰，促进新技术、新组织形式、新产业集群形成和发展，创建2个以上省级绿色工厂(园区)、30家以上智能制造示范车间。

着力打造区域创新高地。实施产业培育、创新人才集聚等六大工程，积极引导企业增加研发投入，力争全社会研发投入占GDP比重达1.5%以上，实现每万人有效发明专利突破2.25件。加快创新平台建设，实施“135”工程升级版，支持高新区打造国家创新型科技园区、衡山科学城与深圳科技园合作打造一流区域性创新高地，新增省级以上工程技术研究中心和重点实验室5家以上。加大对中小企业创新支持力度，加强知识产权保护和运用。推进高新技术产业强基工程三年行动计划，培育发展一批创新引领型企业、平台、机构，加大力度引进一批重点新型研发机构和孵化机构，新增高新技术企业80家以上。全面落实人才新政，引进培育一批高水平创新创业人才和紧缺急需专业技术人才。

积极扩大有效投资。发挥投资关键作用，紧扣国、省规划和重大战略，加大关键领域和薄弱环节有效投资强度。大力实施“1113”重大项目推进计划，加快比亚迪智能制造产业园、建滔电子信息产业园、湘南纺织产业基地、华泰汽车等重大产业项目建设，确保泛亚绿洲铜杆加工、豪曼新能源、稀土高强铝合金、奥瑞克电梯、九明生物等项目建成投产，力促特变电工南方智能电网产业园、界牌陶瓷产业园等项目部分投产。加快推进电网升级改造三年行动计划，力争再造一个衡阳电网。切实提高园区亩均投资、产值和税收。进一步创新投融资模式，加大项目策划包装力度，充分激活民间投资，力争民间投资占比达65%以上。

提质发展现代服务业。聚焦重点企业培育、重点项目实施、重点园区建设、重点品牌打造，推动服务业与先进制造业融合，大力发展信息、科创、商务、人力资源等生产性服务业，促进教育、育幼、养老、医疗、文化、会展等新兴服务业发展。编制全市物流业中长期发展规划，加快京东、工惠驿家、铁路口岸综合物流园等项目建设。创造条件打造临空经济产业园。做大做强现代金融业，吸引更多优质金融机构来衡设立分支机构，实现“一县两行”县域全覆盖。鼓励企业利用电子商务转型升级，加强农村和农产品电子商务应用，健全村级电商体系，力争全市电商交易额突破200亿元。深化“旅游重点项目建设三年行动”，重点抓好华侨城、绿地、梦东方等重大项目建设;力争南岳国家全域旅游示范区创建工作首批通过验收，创建国家4A级旅游景区1家，3A级旅游景区3-5家，旅游接待总人次、旅游总收入分别增长12%、13%以上，加快打造千亿级旅游产业。

(二)坚定不移打好三大攻坚战

强化问题导向和底线思维，举全市之力打硬仗、拔硬寨，以钉钉子的精神打好三大攻坚战。

坚决打好防范化解重大风险攻坚战。严守政府性债务红线，强化政府债务限额管理与预算管理，规范政府举债行为，坚决杜绝变相融资举债，积极消化存量债务，确保隐性债务总量不增加。推动融资平台公司市场化转型，按要求完成市县融资平台公司整合任务。发展普惠金融、科技金融、供应链金融，支持企业债券、公司债券、上市、新三板、股权融资。强化互联网金融和非法集资风险排查和预防预警，加强对地方金融组织全过程监管，严防严打非法集资等违法违规金融活动，坚决守住不发生区域性金融风险的底线。

坚决打好精准脱贫攻坚战。把继续攻坚和成果巩固摆在同等位置，力争3.96万贫困人口脱贫，实现除一类低保兜底对象外贫困人口全部脱贫，全市贫困人口发生率下降到0.36%。进一步加大产业扶贫力度，深入实施就业、基础设施扶贫，坚持扶贫与扶志、扶智相结合，激发贫困地区和贫困群众的内生动力。全面落实教育、健康、住房等扶贫政策，加强扶贫资金管理。研究解决收入水平略高于建档立卡贫困户的群体缺乏政策支持等新问题，不断提高扶贫的满意度和实效性。

坚决打好污染防治攻坚战。加快国家生态文明先行示范区建设，持续优化生态功能布局。实施最严格的环境准入制度，帮助企业制定环境治理解决方案，全面完成突出环境问题整改。持续打好蓝天、碧水、净土三大保卫战和七大标志性重大战役，继续发动污染防治“夏季攻势”，扎实抓好中央环保督察及“回头看”反馈问题整改。大力推进大气污染防治联防联控，加强对工业企业无组织排放、建筑施工扬尘、餐饮企业油烟等方面的重点管控，限期关停取缔淘汰类“散乱污”企业，确保城区空气质量优良率继续保持在80%以上。启动实施湘江保护和治理第三个三年行动计划，深化河长制工作，统筹推进“一江四水”保护与治理，加大水口山、合江套及蒸水不达标水体等重点区域环境综合整治力度，加快县乡黑臭水体整治，推进乡镇饮用水水源地保护，加快城市污水管网建设，完成一半以上乡镇污水集中处理设施建设，确保湘江干流及支流水质持续向好。推进土壤污染综合防治先行区建设。全面整治历史遗留尾矿库，加快关闭矿山生态修复。

(三)凝心聚力推动改革开放再出发

坚持以改革促动破局，以开放搞活发展，敢闯敢干、攻坚破难，推进更深层次改革和更高水平开放。

争当承接产业转移“领头雁”。抢抓湘南湘西承接产业转移示范区建设机遇，对接北上杭、对接粤港澳大湾区，探索完善产业链专题招商、股权招商、基金招商方式，发挥异地商会作用，扎实开展“迎老乡、回故乡、建家乡”等活动，围绕重点产业、重点企业开展强链补链延链招商行动，吸引“三类500强”企业来衡投资，力争引进世界500强企业8家以上，实际到位内资突破500亿元、增长12%。各县(市)区力争每年引进5个以上亿元产业项目，1个以上“三类500强”企业项目，各市直园区引进项目的个数和规模在县(市)区基础上实现翻番。鼓励跨国公司、国内500强企业来衡设立区域总部，建立整合贸易、物流、结算等功能的营运中心。健全项目履约考核管理办法，整治招商引资违约失信行为。进一步做实做强衡阳深圳工业园。

稳步推进重点领域改革。认真落实“巩固、增强、提升、畅通”八字方针，深化供给侧结构性改革，持续巩固“三去一降一补”成果，进一步破除旧动能、培育新动能。如期完成政府机构改革任务，优化政府机构设置和职能配置。加快国企国资改革，积极推进混合所有制改革，做强做优国有资本。理顺城区、园区管理体制机制，加大简政放权力度，充分调动城区、园区的发展积极性。抓紧推进中心城区区划调整。深化综合行政执法体制改革，提升城市管理科学化、精细化、智能化水平。落实个人所得税改革等更大规模的减税措施。深化政府采购、政府购买服务等改革，统筹推进事业单位、财税金融等领域改革。

推动形成全面开放新格局。深度融入“一带一路”建设，推进新引进外向型实体企业、外贸综合服务、跨境电商项目，加强出口示范基地建设和出口品牌建设。推动出口市场多元化，抓好重点企业、重点园区、重点县(市)的外贸业绩增长，引导扶持中小企业开拓国际市场，力争实际利用外资15.6亿美元，增长10%。强化综保区等平台作用，做好航空口岸申报和铁路口岸建设工作。积极发展同发达国家、先进地区友好城市关系，深入推进经贸、文化、教育、中医、体育、旅游等领域合作。

(四)全面实施乡村振兴战略

以乡村振兴战略为总抓手，全力推进农业全面升级、农村全面进步、农民全面发展。

深化农业供给侧结构性改革。推动藏粮于地、藏粮于技落实落地，坚守耕地保护红线，建设高标准基本农田1043万亩，推进全国首批第三代杂交水稻创新基地建设，稳步提升粮食生产效益。完善农村宅基地制度，抓紧出台农村宅基地节约集约利用奖励政策。加快农村集体产权制度、现代农业发展体系等重点改革，持续推进“两权”抵押贷款。着力增加优质绿色农产品供给，加快农产品质量安全追溯体系建设，推动农业发展由增产向提质转变。深化供销合作社综合改革。加强对农村土地流转的规范管理和服务引导，建立健全农村产权(流转)交易市场。发展多种形式的新型农业经营主体。扎实推进农村土地和集体未承包到户资源股份制合作，发展壮大农村集体经济，力争有经营收入的村达80%以上。

推进农村产业兴旺。深入实施“8135”发展战略，大力发展农产品精深加工，延伸产业链条，打造千亿级农副产品种植加工产业。大力实施品牌强农，持续创建“三黄两茶”品牌，新增油茶林30万亩，加快建设“一馆三中心”，叫响“高端油茶看衡阳”品牌，加快打造中国油茶第一强市。抓实农业“三个百千万”工程，发展“一县一特”“一乡一业”，扶持打造区域公用品牌、特色农产品品牌，每个优势产业集中打造1-2个主打品牌，创建省级现代农业特色产业示范园10个。新获证绿色食品、有机产品40个，创建1个以上国家农产品地理标志产品，打造十大领军企业、30个成长型企业。创建一批国家级、省级农业产业强镇和农村一二三产业融合发展试点县，打造一批有特色、上规模、效益好、带动强的休闲农业企业。落实促进农民收入持续较快增长三年行动计划，确保农民稳定增收。

改善农村人居环境。开展“千村整治、百村示范”创建活动，推进农村环境整治“五大工程”和“四好农村路”建设，完成环境综合整治行政村500个，创建美丽乡村示范村33个，加快雨母山、茶山坳等片区建设，打造美丽乡村升级版。推动村规、土规两规合一，力争村庄规划编制覆盖率达100%。深化农村“厕所革命”，改建卫生厕所31万户。抓好第二次全国农业污染源普查。打好农村面源污染防治攻坚战，完善生活垃圾收运体系;扎实开展秸秆禁烧专项行动，推进农作物秸秆综合利用和畜禽养殖废弃物资源化利用。加强传统村落保护。实施“明靓雁城”乡村亮化工程。全面开展“五边”造林，建设一批森林县城、森林乡镇和森林村庄。

加强农村基层基础工作。实施公共服务水平提升工程，推动城镇和乡村基础设施互联互通，加快优质教育、文化、医疗等向农村延伸。关心关爱基层干部，加强农村人才队伍建设，提升“三农”服务能力。完成自然村通水泥(沥青)路1680公里，抓好农村公路窄路加宽和安防工程建设。推进农村水利工程建设。加快农村通讯设施改造，实现所有行政村4G网络全覆盖。健全自治、法治、德治相结合的乡村治理体系。完善农村社区建设。加强农村精神文明建设，培育文明乡风、良好家风、淳朴民风。

(五)统筹抓好区域协调发展

优化城区发展布局，提升县域发展支撑，着力补齐城镇基础设施短板，构建中心城区带动、县域多极支撑、乡镇协同并进的发展格局。

提升中心城区功能品位。推进城市总体规划修编。加快来雁新城、滨江新区、陆家新区、酃湖高铁新城等片区开发。坚持以“TOD/SOD”开发模式和城市经营为导向，大力实施城市“内外畅通”工程，力争比亚迪胶轮有轨电车示范线一期建成投用，启动北三环、长丰大道北延、红湘北路延伸等重点工程，提质更新蔡伦大道北段、立新大道西延等8条道路，打通一批断头路、堵点路，优化道路交通组织。加大城市“双修”力度，成片示范推进旧城改造，加快实施“三清三建”升级版。持续推进农贸市场提质改造。发展装配式建筑、绿色建筑，鼓励有条件的老旧住宅加装电梯。完成“三江六岸”规划管理立法工作，加快“三江六岸”生态绿地建设;实施园林绿化提升工程，推进城市绿地提质改造，力争城市建成区绿化覆盖率达到41%。完成廖家湾变电站110kV电缆隧道建设。保障城市供水供气安全。完善城市洪涝、地质灾害防御体系。加快推进违法建设“五年清零”行动;依法依规逐步化解城建领域积案。加快城区智能停车系统建设，逐步解决城市停车难问题。巩固电动摩托车管理成效。持续优化“两站一场”、学校、医院周边环境。

做强县域经济发展支撑。着眼于建设在全省乃至全国都具有强大竞争力的县域经济强县，支持各县(市)区发展特色产业，打造特色鲜明、竞相发展的区域板块。发展壮大城区经济，促进现代生产要素向中心城区集聚。鼓励衡南县发展高端制造和临空经济等产业;推动衡阳县加快西渡高新区和界牌陶瓷工业园发展;引导衡东县、衡山县、南岳区协同推进生态文化旅游产业发展，打造绿色发展示范高地;支持祁东县做大做优归阳工业园;加快常宁市两大千亿级产业发展;帮助耒阳市加快传统产业转型升级，培育壮大新兴动能。

完善区域综合交通网络。围绕打造“布局合理、衔接顺畅、保障有力、安全高效”的综合交通网络，完成南岳机场站坪扩建工程，启动二期项目前期工作,实现通航城市30个、旅客吞吐量突破100万人次;建成大浦通用机场，打造“全省示范、全国一流”的通用航空产业基地;加快铁路枢纽改造工程前期工作，力争湘桂铁路城区段外迁取得实质性进展，推进安张衡铁路项目前期工作;加快祁东归阳千吨级码头一期工程、湘江衡阳至株洲二级航道建设，启动湘江永州至衡阳三级航道改扩建;推进G107等干线公路建设改造、祁东归阳至常宁蓬塘高速、S337泉溪大桥危桥改造、衡阳至祁东快速干线、南岳高速萱洲互通等项目建设,启动耒云快线前期工作;加快交通信息平台建设;加快道路运输企业转型升级和城乡客运一体化。

(六)打造一流营商环境

以自我革命的勇气推进流程再造、破除发展梗阻，用真心、谋真招、出真力，千方百计为企业解难题，为民众添便利。

提升政务服务环境。全面深化“最多跑一次”改革，深入推进“互联网+政务服务”建设，加快审批服务“四办”改革，着力打破部门间的“信息壁垒”，确保依申请类事项网上可办率达95%以上。鼓励有条件的园区推行不见面式审批服务。深化商事制度改革，抓实政府部门涉企信息统一归集公示工作，强化事中事后监管。深化简政放权，推进审批服务事项办理流程标准化，推行建设领域行政审批制度改革2.0版，清理、精简行政审批事项和涉企收费，规范中间环节和中介组织行为，力争企业开办时限压缩到5个工作日，行政审批事项精简率达75%。全面取消影响群众和企业办事的各类不合法、不合理证明。

大力支持民营经济发展壮大。坚持“两个毫不动摇、三个没有变”，全面落实支持民营经济发展的各项政策措施，在市场准入、审批许可、经营运行、招投标等方面为民营企业创造公平竞争的制度环境。落实各项减税降费措施，进一步减轻企业发展负担。坚持实事求是原则，在行政执法过程中避免简单化、“一刀切”。构建亲清新型政商关系，深入开展“一企一策”帮扶活动，严厉惩处对企业乱检查、乱收费等违纪违法行为。营造法治化制度环境，保护民营企业家人身安全和财产安全，增强企业家信心，让民营企业在市场经济浪潮中尽显身手。

促进生产要素优化配置。建立健全普惠性政策，推动用地、排放、能耗、环境容量等指标向符合国家产业政策和环保要求的重大产业项目倾斜。做大做强担保公司，充分发挥金融信用信息服务职能，用好用活产业投资基金，着力解决企业融资“难贵慢”等问题;优化融资结构和信贷结构，提高直接融资比重，让金融回归服务实体经济本源。扎实推进城乡建设用地增减挂钩工作。积极争取工业直购直供用电、天然气井口气价等政策指标覆盖工业园区。

(七)加快文化强市建设

坚持经济发展与文化繁荣并重，文化产业与文化事业并举，打造更具品质的区域性文化导向型城市。

强力推进全国文明城市创建。坚持把创建全国文明城市作为“一号工程”，加大单项城市荣誉品牌创建力度。深入开展社会主义核心价值观教育，推动习近平新时代中国特色社会主义思想深入人心。大力弘扬中华优秀传统文化、红色文化、社会主义先进文化，推动物质文明和精神文明协调发展。积极创建国家历史文化名城，统筹保护与利用的关系，抓紧推进历史文化街区整修、文化遗产保护、历史文化挖掘、人文环境建设等“四大工程”，让广大群众在优美环境中感受历史文化之美。实施“相约文明”市民素质提升工程，广泛开展身边好人、最美人物、时代楷模、优秀志愿者等典型选评和宣传活动，大力弘扬文明新风。

完善公共文化服务体系。扎实推动公共文化服务标准化均等化发展。推进行政村综合性文化服务中心和中央广播电视节目无线数字化覆盖工程。加快图书馆总分馆服务体系建设，推动全民阅读。建成影剧艺术中心、青少年活动中心、市图书馆，启动市博物馆、科技馆、规划馆建设。继续开展文化惠民活动，完成送戏下乡1010场、送电影下乡5万场，助推公共文化“进村入户”。强化文化娱乐、新闻出版、广播影视等市场监管，加强网络空间治理和内容建设，不断净化文化市场环境。

大力发展现代文化产业。引进培育骨干文化企业。壮大新闻出版、广播影视等传统文化产业，积极发展新型文化业态。大力发展“文化+”，深入挖掘文化内涵，促进文化与旅游、农业、科技等领域融合发展。进一步繁荣文艺创作，力争电影《援军明日到达――1944衡阳保卫战》成功开拍。推进本土特色文化传承发展，举行“纪念王船山诞辰400周年”系列活动。

(八)全面保障和改善民生

坚持尽力而为，量力而行，聚焦老百姓最盼最急最忧最怨的问题，一件接着一件办，一年接着一年干，全面完成各项民生实事任务，切实为群众谋福祉。

提高就业质量和居民收入水平。实施就业优先政策，确保城镇登记失业率控制在4.5%以内。实施创新创业带动就业工程，培育一批创业孵化基地。重点抓好高校毕业生就业创业，吸引在衡高校毕业生扎根衡阳。继续实施“雁归兴衡”行动计划，促进返乡农民工创业。拓宽退役军人、去产能分流职工、农村转移劳动力、城镇就业困难人员等群体就业渠道。抓好农民工工资清欠保支。落实就业扶贫政策，确保就业扶贫三年行动计划如期完成。

完善社会保障体系。强化兜底保障功能，落实基本养老保险中央调剂金制度，持续扩大各项社会保险覆盖面。实施统一的城乡居民基本医疗保险、大病保险和医疗救助制度，加快推进异地就医联网直接结算。健全最低生活保障、特困人员供养和临时救助制度，完善社会救助体系。健全农村“三留守”人员关爱服务体系，切实保障妇女、儿童、老年人、残疾人等弱势群体权利。完成1.21万套“两房两棚”改造任务。坚持“房子是用来住的，不是用来炒的”定位，构建房地产市场健康发展长效机制，完善住房市场体系和住房保障体系。

发展公平而有质量的教育。持续加大教育经费投入。推进标准化学校建设，新建、改扩建义务教育学校63所，新增学位7.8万个，加快消除大班额。推动城乡义务教育一体化发展，完成“全面改薄”任务。高质量发展公办教育，加快铁一中初中部、市十七中、市七中迁建和市华新实验中学改扩建等项目建设;推进居民小区配套规划建设中小学校、幼儿园，实施第三期学前教育行动计划。规范社会力量兴办教育，鼓励和支持社会各界特别是企业发展职业教育。加快衡南县、衡山县、衡东县特殊学校建设。全力推进衡阳幼师高专、市示范性综合实践基地建设。推动市城区教育信息化建设项目，启动市直教育信息化工程。继续做好进城务工人员随迁子女受教育工作。扎实抓好家庭教育、心理健康教育等工作，提高学生身心素质。实施乡村教师支持计划，加强师德师风建设，培养高素质教师队伍。

推进健康衡阳建设。深化医药卫生体制改革，大力推进医联体建设，完善分级诊疗体系，提高家庭医生签约服务工作质量，打造湘南区域性医疗中心。落实国家基本药物制度，加强基本药物供应保障。促进生育政策和相关配套政策衔接，加大对计划生育家庭扶助力度，落实优生优育、出生缺陷防治等生殖健康服务，提升妇幼保健服务能力。推进医疗卫生与养老服务相结合。健全医疗纠纷防范和处理机制，促进医患和谐。发挥省运红利，完善公共体育设施，提升体育场馆经营能力和体育产业发展水平。加快市体校搬迁，大力发展社区体育，广泛开展全民健身运动。

打造共建共治共享的社会治理格局。推进应急管理体制改革，落实地方党政领导干部安全生产责任制，强化企业主体责任，着力堵塞监管漏洞，统筹防范自然灾害、食品药品安全、社会舆情等方面的风险，坚决防范和遏制较大以上事故发生。深入推进“扫黑除恶”专项斗争和新一轮禁毒人民战争。强化社会治安立体化防控体系建设，全面落实“一村一辅警”制度，打造“衡阳快警”空地网一体化巡防体系，完成“雪亮工程”建设任务，着力打造“平安衡阳”升级版。加强民族宗教工作。深化阳光信访、责任信访、法治信访建设，开展重点领域专项治理，维护群众合法权益。

大力支持国防和后备力量建设。推动军民科技基础要素融合，加快军民融合产业项目建设，争创国家军民融合创新示范区。加强退役军人事务管理，抓好新形势下全民国防教育，强化新时代国防动员和后备力量建设，做好民兵预备役和人防工作，巩固军政军民团结的良好局面。

三、锻造最强执行力政府

坚持把讲政治、重执行、创一流作为政府工作的生命线，大力推行“最强执行力建设年”行动，打造人民满意的服务型政府。

(一)加强政治建设。始终坚持党对一切工作的领导，始终坚持以习近平新时代中国特色社会主义思想为指导，增强“四个意识”，坚定“两个维护”，严守政治纪律和政治规矩，坚决贯彻落实中央方针政策、省委省政府和市委工作部署，确保政令畅通、令行禁止。坚持全面从严治党政治责任与政府工作责任一起落实，扎实开展“不忘初心、牢记使命”主题教育，坚定理想信念，以好的作风确保好的成效。

(二)提升履职本领。牢固树立新发展理念，以思想观念的更新推动领导方式、工作方式和发展方式转变。大兴学习和调查研究之风，在学习和实践中找思路、想办法。针对干部本领恐慌、经验盲区、能力弱项，开展精准培训，增强抓经济工作的专业知识、专业素养、专业精神，提升创造性贯彻落实的能力和水平。激励干部担当作为，突出“干”字为先，推行“马上就办”。对敢担当、善作为的干部放心地用，对不作为、慢作为、乱作为的干部坚决地调，对符合“三个区分开来”的过失过错大胆地容，真正让干部把心思用在抓发展、快落实上。

(三)坚决依法行政。牢固树立法治理念，严格依照宪法法律行使职权、履行职责、开展工作，开门接受人大及其常委会的法律监督、工作监督，自觉接受政协的民主监督，认真接受监察监督和审计监督，主动接受社会舆论监督，切实做到“法定职责必须为，法无授权不可为”。加强行政法规规章立改废释工作，全面推行规范性文件合法性审查制度。严格规范政府行为，落实重大行政决策程序，坚持公正文明执法，健全完善行政执法体制。持续加大政务公开力度，动态推行权力清单、责任清单、负面清单制度，让权力在阳光下运行。建设诚信政府、法治政府，依法依纪妥善处理历史遗留问题，决不搞“新官不理旧账”。

(四)坚守廉洁底线。认真履行党风廉政建设主体责任和“一岗双责”，严格执行“八项规定”和廉洁从政各项规定。强化公共权力运行、公共资金、公共资产、公共资源交易、公共工程监管，最大限度降低制度性成本、压缩权力寻租空间。深挖、彻查、严打涉黑涉恶“关系网” “保护伞”，严禁利用领导干部职权或职务上的影响“提篮子”、谋私利，坚决斩断破坏市场公平的“权力黑手”，坚决惩治各类腐败行为。加强廉政教育和道德修养，筑牢拒腐防变思想防线，让每一名政府工作人员都干干净净为人民做事。

各位代表!幸福需要奋斗，实干成就未来。让我们更加紧密地团结在以习近平同志为核心的党中央周围，在省委、省政府和市委的坚强领导下，不忘初心、牢记使命，开拓奋进、埋头苦干，加快建设名副其实的省域副中心城市和最美地级市，当好承接东部产业转移“领头雁”，以优异的发展业绩庆祝新中国70华诞!
